# Blood Immune Cell Biomarkers in Lung Cancer Patients Undergoing Treatment with a Combination of Chemotherapy and Immune Checkpoint Blockade

**DOI:** 10.3390/cancers14153690

**Published:** 2022-07-28

**Authors:** Miriam Möller, Steffi Turzer, Georgi Ganchev, Andreas Wienke, Wolfgang Schütte, Barbara Seliger, Dagmar Riemann

**Affiliations:** 1 Clinic of Internal Medicine, Hospital Martha-Maria Halle-Dölau, D-06120 Halle, Germany; miriam.moeller@martha-maria.de (M.M.); georgi.ganchev@martha-maria.de (G.G.); wolfgang.schuette@martha-maria.de (W.S.); 2 Institute of Medical Immunology, Martin Luther University Halle-Wittenberg, D-06112 Halle, Germany; steffi.turzer@uk-halle.de (S.T.); barbara.seliger@uk-halle.de (B.S.); 3 Institute of Medical Epidemiology, Biostatistics, and Informatics, Martin Luther University Halle-Wittenberg, D-06112 Halle, Germany; andreas.wienke@uk-halle.de

**Keywords:** biomarker, immune checkpoint blockade, dendritic cells, immune monitoring, lung cancer, prognosis

## Abstract

**Simple Summary:**

Tumor cells can evade destruction via immune cells by expressing coinhibitory membrane molecules, which can suppress tumor-specific T cells. Immune checkpoint inhibitor therapies act by blocking these inhibitory pathways via monoclonal antibodies. Although this type of immunotherapy has shown promising results for advanced cancers of different entities during recent years, an important challenge is to identify the baseline characteristics of patients who will mostly benefit from such treatment. Blood biomarkers have limitations to reflect the tumor microenvironment but are easier to handle than markers in tumor lesions. The aim of our study was to identify blood cell parameters correlating with patients’ survival in 90 patients with non-small cell lung cancer undergoing immune checkpoint inhibitor therapy combined with chemotherapy. We found that patients with a neutrophil-to-lymphocyte ratio ≥6.1, a percentage of HLA-DR^low^ monocytes ≥22%, a frequency of slan+ non-classical monocytes <0.25%, and/or of dendritic cells ≤0.14% of leukocytes had a poorer prognosis. Long-term survivors were patients without any of the risk factors investigated. Our results implicate that blood neutrophil counts, special types of monocytes, and the number of blood dendritic cells might be useful predictive biomarkers for cancer patients’ survival.

**Abstract:**

Although immune checkpoint inhibitor (ICI) therapies have improved the treatment of patients with advanced non-small cell lung cancer (NSCLC), several patients do not achieve durable clinical responses. Biomarkers for the prediction of therapy responses are urgently needed. To identify blood cell parameters correlating with patients’ survival, immune cells from 90 patients with NSCLC undergoing a combination of ICI and chemotherapy were prospectively monitored. At the time point of the first and third antibody administration, complete leukocyte blood count, the percentage of HLA-DR^low^ monocytes, the percentage of 6-Sulfo LacNAc (slan)+CD16+ non-classical monocytes, and the number of circulating dendritic cell (DC) subtypes, as well as T-, B-, and NK cells were determined by multi-color flow cytometry in peripheral blood. The prognostic value of the immune cell parameters investigated was evaluated by patients’ survival analysis, with progression-free survival (PFS) as the main criterion. A total of 67 patients (74.4%) showed a partial remission or a stable disease, and 35% of patients even survived 12 months and longer. Patients with a neutrophil-to-lymphocyte ratio (NLR) ≥6.1, a frequency of HLA-DR^low^ monocytes ≥22%, of slan+ non-classical monocytes <0.25% of leukocytes, and/or a sum of myeloid DC (MDC) and plasmacytoid DC (PDC) ≤0.14% of leukocytes had a poorer prognosis. The hazard ratio for PFS was 2.097 (1.208–3.640) for the NLR, 1.964 (1.046–3.688) for HLA-DR^low^ monocytes, 3.202 (1.712–5.99) for slan+ non-classical monocytes, and 2.596 (1.478–4.56) for the MDC/PDC sum. Patients without any of the four risk factors showed the best PFS. Furthermore, low NK cell counts correlated with shorter PFS (cutoff 200 cells/µL). Female patients had lower baseline NK cell counts and a shorter PFS. Our study confirms the usefulness of blood immune cells as biomarkers for clinical response and survival in NSCLC patients undergoing a combined ICI/chemotherapy.

## 1. Introduction

Lung cancer is one of the most common malignant tumors and a leading cause of cancer-related death worldwide [1]. Non-small cell lung cancer (NSCLC), accounting for about 83% of all patients with lung cancer, is subdivided into adenocarcinoma (AC) (50–70%), squamous cell carcinoma (SqC) (20–30%), and other subtypes (<10%) [2]. At the time point of diagnosis, about 75% of NSCLC patients have an advanced disease associated with a bad prognosis implying low survival rates [3]. Platinum-based chemotherapy has been the standard therapy despite modest responses to these agents and short intervals until disease progression [4,5]. Recently, immune checkpoint inhibitors (ICI) targeting the PD-L1/PD-1 signaling axis have emerged as a treatment option for these patients, although only a limited proportion of patients benefit [6,7]. The identification of this target population remains challenging, which denotes an unmet need to develop accurate biomarkers predictive of response to immune checkpoint inhibition for patients’ selection.

In cancer patients, tumor-specific immune responses are inhibited, and in patients with advanced lung cancer, a systemic immune suppression has been observed [8]. Different cells and factors have been implicated in this process, including regulatory T cells, myeloid-derived suppressor cells (MDSC), various soluble factors, and cytokines as well as inhibitory receptor molecules expressed by tumor cells [9]. Despite blood cell biomarkers’ difficulty in reflecting the tumor microenvironment, immune cell profiling in peripheral blood is an attractive alternative tool for biomarker identification. The aim of the current study was to investigate blood immune cells as putative biomarkers to select patients who could benefit from immune/chemotherapy. We enrolled patients with histologically confirmed unresectable locally advanced or metastatic NSCLC lung cancer prior to PD-1 or PD-L1 blockade treatment combined with chemotherapy. Since a high neutrophil/lymphocyte ratio (NLR), a high number of HLA-DR^low^ monocytes, and low DC levels correlated with a bad patients’ survival in a recent study with lung cancer patients undergoing ICI monotherapy [10], we mainly focused on these three risk factors in our study. We complemented the blood biomarkers by 6-Sulfo LacNAc (slan)+CD16+ non-classical monocytes, since these cells have been shown to be involved in anti-tumoral activity [11].

## 2. Materials and Methods

The study was approved by the institutional review board of the Ärztekammer Sachsen-Anhalt (No. 69/18). EDTA peripheral blood samples were obtained from patients with advanced lung cancer of NSCLC histology. From June 2019 to June 2021, 90 patients with histologically confirmed unresectable locally advanced or metastatic lung cancer prior to PD-1 or PD-L1 blockade treatment in combination with chemotherapy were prospectively enrolled (convenience sample). Patients met the following criteria: age >18 years, histologically confirmed diagnosis of advanced lung cancer, adequate organ function, and capacity to make an informed decision. All patients were negative for epidermal growth factor receptor mutation or anaplastic lymphoma kinase translocation. Furthermore, patients with a previous history of active autoimmune disease were excluded. All patients gave written informed consent for the study proposal and procedures. The cutoff date of the study was January 2022.

Patients with SqC received a combined immunochemotherapy with nab-paclitaxel, carboplatin, and pembrolizumab, according to KEYNOTE-407 trial [12]. The same regimen was also given to patients with thyroid transcription factor (TTF)-1-negative AC, as pemetrexed might be less effective in these patients [13]. Since, according to the IMpower150 study, the combination has a clear advantage in the presence of liver metastases [14], patients with AC and liver metastases received a combined immune/chemotherapy with atezolizumab, bevacizumab, carboplatin, and nab-paclitaxel. All other patients with AC received a combined immunochemotherapy with pemetrexed, carboplatin, and pembrolizumab according to the data from the KEYNOTE-189 trial [15]. Furthermore, four patients received thorax radiation before or during the initiation of the systemic therapy due to a high tumor burden with existing or threatening superior vena cava syndrome. With ongoing maintenance therapy, some patients also received additional radiation therapy either due to particularly good tumor response and oligometastasis to improve the prognosis according to the study by Gomez et al. [16,17] (7 patients), or in terms of palliative radiation, for example, in the case of pain or symptomatic tumor progress (10 patients).

Patients’ responses were determined according to the Response Evaluation Criteria in Solid Tumors (RECIST 1.1). Patients underwent CT scans at baseline and after 10 weeks. Subsequent assessments of disease extent by CT scan were scheduled every 12 weeks or earlier if clinically indicated. In the case of progressive disease, patients were allowed to continue the treatment if clinical improvement was maintained, and CT was repeated after 8 weeks to confirm progression. Besides RECIST-defined objective response, we assembled complete and partial clinical remission with stable disease to obtain the disease control benefit group, which was compared to the patients’ group without durable clinical benefit. Primary endpoint was the progression-free survival (PFS) of patients. PFS was defined as the time elapsed from initiation of ICI/chemotherapy until the first observation of progressive disease or death from any cause. Overall survival (OS) was defined as the time from initiation of ICI/chemotherapy until death from any cause. Patients who did not die or progress and those lost to follow-up were censored.

Peripheral blood samples were collected within 7 days before initiation of ICI/chemotherapy (time point 1, baseline) and prior to the third cycle of ICI therapy (time point 2). In case of early treatment drop-out before the expected time point 2, a peripheral blood sample was drawn if possible before the first assessment of disease response. The leukocyte count and the complete blood count were determined using a CELL-Dyn Ruby (Abbott Lab., Wiesbaden, Germany). Circulating DC subpopulations were identified with the “Blood DC Enumeration Kit” (Miltenyi, Bergisch Gladbach, Germany) supplemented for gating reasons with CD45 APC-H7 and an HLA-DR V500 monoclonal antibody (mAb) (BD Biosciences, Heidelberg, Germany). Briefly, 300µL whole blood was incubated with a cocktail of mAbs including anti-CD1c PE as a marker for myeloid DC (cDC2), CD141/BDCA-3 APC (myeloid cDC1), and CD303/BDCA-2 FITC for plasmacytoid DC (pDC) [18]. The test kit contained an anti-CD14 mAb and CD19 PE-Cy5 to exclude monocytes and B cells from the analysis and a dead-cell discriminator. After antibody incubation, erythrocyte lysis, and two washing steps, blood cells were fixed according to the manufacturer’s instructions. At least 1 million blood leukocytes were analyzed. The gating strategy is illustrated in Figure S1. HLA-DR expression on monocytes was quantified using a mAb labeled on a protein/fluorophore ratio of 1/1 (clone L243; QuantiBRITE™ reagent; BD Biosciences). A total of 50µL of blood was stained according to the manufacturer’s instruction. A standard curve for antigen quantification was established using multi-level calibrated QuantiBRITE beads (BD Biosciences). The measured geometric mean fluorescence intensity (MFI) of the gated population was converted into “antibody molecules bound per cell” (ABC) using a Microsoft Excel™ spreadsheet (version 2016, Microsoft Corporation, Redmond, WA, U.S.). HLA-DR MFI values of ≤5000 ABC for the whole monocyte population have been designated as “immunoparalysis” in former studies, since the patients are at high risk of infectious diseases [19]. Taking an MFI of 5000 ABC as a borderline value for a low HLA-DR intensity, the number of HLA-DR^low^ monocytes was estimated as a percentage of monocytes, as described in [20]. A lysed whole blood technique with 8-color staining of blood cells was used for the immune cell labeling of lymphocytes and monocytes. A sample of 300 µL of EDTA-treated blood was subjected to staining with mAbs specific to slan (M-DC8) FITC (Miltenyi Biotec); CD56 PE from Beckmann Coulter (Hamburg, Germany); CD16-PE-Cy7 from Biolegend (San Diego, CA, U.S.); and CD19 PerCP-Cy5.5 from InVitrogen (Thermo Fisher, Waltham, MA, U.S.); all other mAbs (CD14 APC, CD45 APC-H7, CD3 V450, HLA-DR V500) were from BD Biosciences. The blood–mAbs mixture was incubated for 15 min at room temperature in the dark before 4 mL of 1:10 FACS erythrocytes lysing solution (BD Biosciences) was added. After 10 min of incubation and two washing steps, cells were analyzed in the flow cytometer. Gating strategy for slan+ non-classical monocytes has been provided in Figure S2. Blood cell samples were measured on a FACS CANTO II Flow Cytometer (BD Biosciences, Heidelberg, Germany). Data analysis was performed using the BD FacsDIVA^TM^ software. Since standardized procedures are essential to allow for inter-individual comparisons in the context of studies persisting several months, Cytometer Setup and Tracking (CST) Beads (BD Biosciences) were used daily to set standardized geometric MFI ranges in the fluorescence channels used.

The statistical analyses were performed with the commercial software SPSS 28.0 (SPSS Inc., Munich, Germany). Medians with interquartile ranges (IQRs) are given for most data. Differences in the immune cell parameters between patient groups or between different time points were analyzed using non-parametric tests for unpaired or paired samples, as appropriate. Accordingly, the comparison between different patient groups was based on the Mann–Whitney U test or the Chi-Square test. Survival analysis comprised a descriptive presentation of the cumulative survival functions according to Kaplan-Meier, and differences among the curves were evaluated using the log-rank test. Univariable and multivariable analyses were performed using the Cox proportional hazards model. Correlations among quantitative variables were based on the non-parametric Spearman rank correlation coefficient. For the primary outcome, a *p*-value of less than 0.05 was considered statistically significant; *p*-values of secondary outcomes were interpreted exploratorily.

## 3. Results

### 3.1. Patient Characteristics and General Outcome

The general baseline characteristics of NSCLC patients of this study are summarized in Table 1. The median age was 65 years (range, 31–87 years); most patients were male (67%) and smokers (90%). There was no relevant difference in survival found between 18 patients with tumor recurrence and 72 patients with primary advanced state; therefore, they were analyzed together. All patients received at least two cycles of anti-PD-1/PD-L1 therapy. Patients treated with pembrolizumab underwent a mean of 9 cycles (range 2–31) and those patients treated with atezolizumab underwent a mean of 10 cycles (range 8–12). The median follow up was 13 ± 2.2 months. As shown in Table 1, most of the patients responded to therapy, but often only for a few months. The rate of confirmed objective response was 74.4% for all patients (75% for AC, 70.4% for SqC). Ten patients stopped treatment before the third antibody application, in most cases due to clinical worsening. Patients without a disease control had a median OS of 4 months (95% CI: 2.8–5.2). At the censoring date, 23 patients (25.6%) were still on treatment. The global median PFS was 14 ± 2.1 months (95% CI: 9.8–18.2) and the median OS 18 ± 1.6 months (95% CI: 14.9–21.1).

### 3.2. Blood Cells and Therapy Response

In order to determine blood biomarkers, which predict patients’ clinical response, the composition of blood immune cells in the patient group “progressive disease/therapy discontinuation” and the group “clinical response to therapy” was investigated (Table 2). In comparison to baseline values, the differences observed between the groups were more pronounced at the third cycle of ICI therapy. At baseline, patients with a clinical response to therapy had lower neutrophil counts and higher numbers of MDC. At the third cycle of ICI therapy, the clinical response group had significantly lower neutrophil counts, a lower NLR, and lower frequencies of HLA-DR^low^ monocytes. Furthermore, higher numbers of slan+ non-classical monocytes and higher frequencies of MDC and PDC were associated with clinical response.

Predictor variables with a significant difference between the patients’ groups with and without a PFS of ≥12 months were analyzed with ROC curves to determine the overall strength of association (area under the ROC curve [AUC]) and the optimal cutoff point for the prediction of therapy response (maximizing the sum of sensitivity and specificity). The consideration of the single parameters NLR and HLA-DR^low^ monocytes, evaluated at baseline, resulted in unsatisfactory AUC values <0.7. The best AUC values of ROC curves were observed both for the baseline parameters slan+ non-classical monocytes (AUC 0.725; *p* = 0.001) and for the sum of MDC/PDC (AUC 0.734; *p* = 0.001) (Table S1). At the time point of cycle 3 of ICI therapy, better AUC values were observed; a lower NLR correlated with long term PFS (AUC 0.749) as did a lower amount of HLA-DR^low^ monocytes (AUC 0.676). Otherwise, higher frequencies of slan+ non-classical monocytes (AUC 0.804) and of the sum of MDC/PDC (AUC 0.817) correlated with long term PFS (Table S1). The cutoff values of the risk factors were >6.1 for the NLR, >22% for the HLA-DR^low^ monocytes, <0.25% of leukocytes for slan+ non-classical monocytes, and <0.14% of leukocytes for the sum of MDC/PDC. Of the 56 patients with the AC histotype, 25 patients had no risk factor (45%), 17 patients had one or two risk factors (30%) and 12 patients had three or four factors (21%). Out of the 27 SqC patients, 11 had no risk factor (41%), 8 patients had one or two factors (30%), and 7 patients had three or four (26 %). At cycle 3, 27 out of 50 AC patients had no risk factor (54%), as had 10 out of 25 SqC patients (40%).

### 3.3. Comparison of Baseline and Third-Cycle Blood Cell Markers

**Figure 1:**
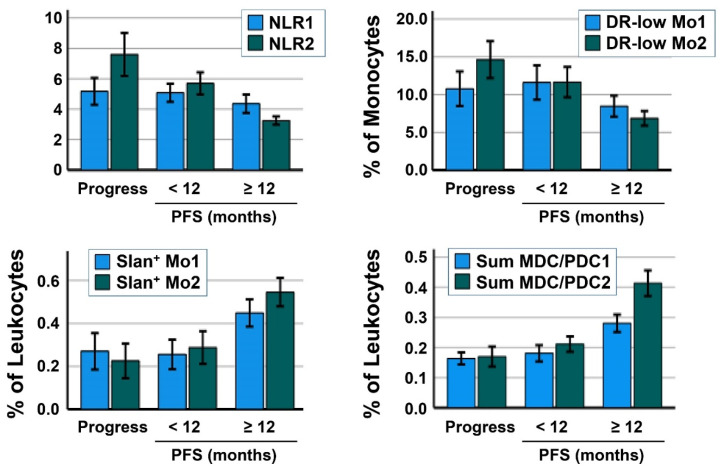
Correlation of blood immune cell markers in the patients’ groups progression/therapy discontinuation, PFS <12 months und PFS ≥12 months overtime (baseline values as 1, ICI-third cycle values as 2). Mean values and error bars are displayed regarding the NLR, the amount of HLA-DR^low^ MDSC (% of monocytes), the percentage of slan+ non-classical monocytes (% of leukocytes), and the sum of MDC/PDC (% of leukocytes).

Investigating immune cell composition overtime, an increase in neutrophil counts could be observed in patients with progress/therapy discontinuation. Otherwise, a decreased neutrophil count was found in patients with a clinical response to therapy (Figure 1). In addition, HLA-DR^low^ monocytes increased with disease progress, whereas a decrease was detected in patients with a PFS ≥12 months. This decrease was associated with lower baseline values in the patients’ group with the best clinical benefit. Both for slan+ non-classical monocytes and the sum of MDC/PDC, no obvious differences were found between patients with progression and short-term PFS. Only patients with a PFS ≥12 months showed an increase of these parameters, although starting from higher baseline values (Figure 1).

### 3.4. Survival Analyses

**Figure 2:**
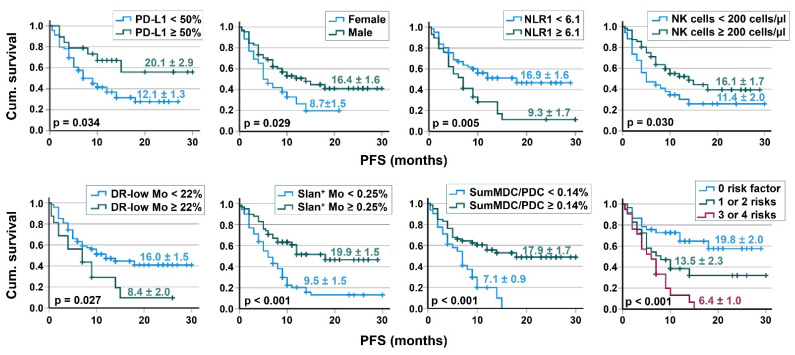
Relationship between risk factors/baseline immune cell parameters and patients’ PFS. Kaplan–Meier curves are shown for PD-L1 expression of tumor lesions, the sex, NLR, NK cells (cells/µL blood), HLA-DR^low^ MDSC (% of monocytes), slan+CD16+ non-classical monocytes (% of leukocytes), the sum of MDC/PDC (% of leukocytes), and a ‘Risk Score’ of the 4 risks ‘high NLR, high HLA-DR^low^ MDSC, low slan+CD16+ non-classical monocytes and low MDC/PDC sum’, with mean survival time and *p* value of the log rank test.

Kaplan–Meier analyses were performed in order to determine whether there were survival differences based on several risk factors, including sex and the immune cell repertoire. Lung cancer patients of the two main NSCLC histotypes AC and SqC had a comparable survival. Patients with ≥50% PD-L1 staining in tumor lesions had a longer PFS (*p* = 0.034, Figure 2) and a tendency towards a better OS (*p* = 0.062). Furthermore, female patients had a shorter PFS (*p* = 0.029) and a tendency towards a worse OS in our study (*p* = 0.078). No relevant difference was found for the smoker status, for patients’ age (<75 and ≥75 years), or for the number of metastases. With respect to the baseline immune cell parameters, 61 patients with a NLR <6.1, 74 patients with a frequency of HLA-DR^low^ <22% of monocytes, 40 patients with ≥0.25% slan+ non-classical monocytes (as % of leukocytes), and 59 patients with a sum of MDC/PDC ≥0.14% of leukocytes showed better PFS compared to the respective reference group, as illustrated in Figure 2. The 21 patients with three or four immune cell risk factors had a worse PFS than the 29 patients with one or two risk factors, which had a worse PFS than patients without any risk factor (Figure 2). No relevant survival differences were found for total lymphocyte counts or for the numbers of T and B cells. With respect to NK cells, 34 patients with <200 NK cells/µL blood had a worse PFS than patients with higher NK cell numbers (Figure 2). Results of univariable prognostic factor analysis (Kaplan–Meier and Cox regression) are provided in Table 3. In a multivariable Cox regression analysis of PFS considering the covariates sex and PD-L1 expression of tumor lesions, the baseline values of NLR, HLA-DR^low^ monocytes, slan+ non-classical monocytes, and the sum of MDC/PDC were independent prognostic factors.

### 3.5. Correlation of Immune Cell Subpopulations

The baseline neutrophil counts directly correlated with the monocyte counts, and were correlated even stronger with the percentages of HLA-DR^low^ MDSC (Table 4). Neutrophil numbers did not correlate with lymphocyte counts). The neutrophil count indirectly correlated with the percentage of CD16+ monocytes, especially with the percentage of slan+ non-classical monocytes and with the sum of MDC/PDC. HLA-DR^low^ MDSC indirectly correlated with CD16+ monocytes, including slan+ non-classical monocytes. Furthermore, an indirect correlation between the frequency of HLA-DR^low^ MDSC and the sum of MDC/PDC was detected (Table 4).

### 3.6. Comparison of Female and Male Patients

**Figure 3:**
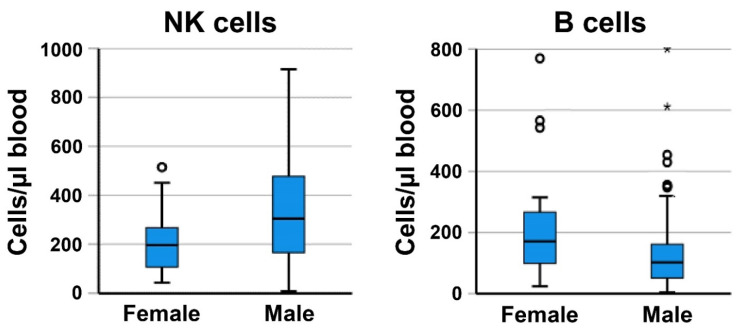
Comparison between baseline values of NK cells and B cells, respectively. Boxplots show a significant difference (*p* < 0.05) between female and male NSCLC patients, with lower NK cell counts and higher B cell counts in female patients. The whiskers indicate the largest/lowest points inside the range defined by 1st or 3rd quartile plus 1.5 times interquartile range (IQR). The circles represent outliers, the stars are extreme outliers (outside triple IQR).

To obtain insight into the observed sex-specific differences in PFS, clinical parameters and immune monitoring results were compared in female and male patients. The frequency of female never-smokers was 23.1%, higher than that in male patients (9.4%). The Sqc histology was rare in female (7.7%) compared to male patients (39.1%). Interestingly, 65.4% of female patients had ≥3 metastases, whereas only 45.3% of male patients were in this risk group. Comparing PD-L1 expression of tumor tissues, only 3/24 female patients (12.5%) expressed ≥50% PD-L1 in the tumor lesions, whereas 16/64 male patients (25%) were in this group associated with a better PFS. No difference was found regarding the age of patients as well as for most of the blood parameters investigated, including the NLR and HLA-DR^low^ MDSC (Table S2). However, whereas female patients had higher baseline amounts of B cells, male patients had higher NK cell numbers, as illustrated in Figure 3. DC subpopulations showed a tendency towards higher values in male patients, significant only with respect to the amount of CD141+ MDC (Table S2).

## 4. Discussion

Lung cancer therapy has been revolutionized by the implementation of ICI therapy. Since not all patients with advanced or metastatic disease can benefit from ICI therapy, predictive biomarkers are an urgent issue [21]. The optimal predictive biomarker should be easily applicable in clinical settings, cost-effective, and provide an accurate prediction of a patient’s clinical response. Currently, the only FDA-approved biomarkers for ICI therapy are PD-L1 expression of tumor tissue, tumor mutational burden, and DNA mismatch repair deficiency/microsatellite instability (for review, see [22]). Blood biomarkers have difficulties reflecting the tumor microenvironment but are easier to handle. Blood-based cellular immune biomarkers are promising in predicting responses to ICI therapy due to specimen accessibility, opportunity for serial monitoring, quantitative measurement, and the availability of the unique analytic platforms [23]. High absolute neutrophil counts as well as a high NLR can identify non-responders to immune checkpoint inhibition; a meta-analysis reported that a high NLR resulted in a worse PFS and OS in NSCLC, melanoma, and genitourinary cancer treated with ICI therapy [24]. First results have been reported also for monocytes; using CyTOF mass cytometry, Olingy et al. described a link of CD33-high classical monocytes to the effectiveness of ICI therapy in NSCLC patients [25]. Using multi-color flow cytometry of blood from NSCLC patients undergoing ICI monotherapy, a high NLR, a high frequency of HLA-DR^low^ monocytes, and low DC percentages were defined as adverse factors for clinical response and patients’ survival [10]. In the current study with 90 NSCLC patients undergoing a combination of ICI and chemotherapy, we confirm the immunomonitoring data of ICI monotherapy and show that the NLR, the frequency of HLA-DR^low^ monocytes, and the sum of MDC/PDC might be useful predictive biomarkers both for the clinical response to therapy and for patients’ survival. We complemented the three biomarkers by slan+CD16+ non-classical monocytes, which show a behavior comparable to DC regarding patients’ survival (PFS and OS), which was significantly improved with higher amounts of slan+ monocytes (cutoff 0.25% of leukocytes) and with a higher sum of MDC/PDC (cutoff 0.14% of leukocytes). Patients without any of the four risk factors (high NLR, high number of HLA-DR^low^ monocytes, low frequency of slan+ non-classical monocytes, and a low MDC/PDC sum) had the best outcome, while patients with three or four risk factors had the worst PFS in this study.

The therapeutic activity of ICI is the result of a complex interplay between multiple factors in the tumor microenvironment and the immune system. Mechanistically, ICI could either compete for the ligands of co-inhibitory receptors or control the surface expression of these receptors. The efficacy of ICI treatment depends on the tumor mutational burden [26], the intratumoral heterogeneity (which is associated with patterns of immune suppression) [27], and the tumor cell expression of immune checkpoint molecules (for review see [22]). Different mechanisms of immune suppression are known to prevent effective anti-tumor immunity, including increased secretion of immunosuppressive cytokines, enhanced differentiation of immune effector cells to a regulatory phenotype, and an influx of MDSC [28]. Currently, considerable efforts are performed to elucidate the mechanisms controlling the development of primary and acquired resistance to ICI therapy [29]. By deciphering the resistance mechanisms involved, strategies might be developed to overcome resistance and treatment failure. ICI combination with chemotherapy improved the therapy response in our patients’ cohort compared to ICI monotherapy in the previous study; 74.4% of the 90 NSCLC patients showed a clinical response compared to 40% of the 35 NSCLC patients undergoing ICI monotherapy [10]. First-line ICI therapy combined with chemotherapy is among the current standard therapies for advanced NSCLC, compensating for the disadvantage of early treatment failure with ICI monotherapy [15]. Several studies have reported that a combination therapy of ICI plus other approaches, such as chemotherapy [30,31] or radiotherapy [32,33], can improve the prognosis of patients. Platinum agents, the backbone of chemotherapy for metastatic NSCLC, can increase antigen presentation by cancer cells, promote T cell trafficking into the tumor microenvironment, and diminish MDSC [34,35]. Chemotherapy has been shown to induce immunogenic cell death, enhance tumor antigenicity, disrupt immune suppressive pathways, and enhance effector T-cell response (for review, see [36]). Intriguingly, expression of immune checkpoint molecules such as PD1 and PD-L1 in the tumor lesions has been linked to patients’ responses to chemotherapy in NSCLC [37].

Neutrophils, representing the most abundant myeloid cells in human blood, are emerging as important regulators of cancer. Neutrophils have been discussed to contain a subpopulation that facilitates tumorigenesis, promotes tumor growth and metastasis, stimulates angiogenesis, and mediates immunosuppression [38]. The NLR combining neutrophils and lymphocytes is an established marker for the prognosis of lung cancer patients in therapy [22,24,39]. Within this ratio, neutrophils seem to be more important than lymphocytes in our study, because for lymphocyte numbers, no significant differences could be detected in patients with and without a response to therapy. With respect to lymphocytic subpopulations, only NK cell numbers had an influence on patients’ prognosis: A lower NK cell number (cutoff 200 cells/µL) correlated with shorter PFS in this study, an observation shared by other authors [40,41]. In our study, high neutrophil numbers positively correlated with monocyte counts and with the percentage of HLA-DR^low^ MDSC, as described earlier in NSCLC patients of different tumor stages [20]. HLA-DR^low^ monocytes are known to suppress the functions of lymphocytes in cancer patients [42,43], similar to the situation described in sepsis [44] and major trauma [45]. We investigated HLA-DR^low^ MDSC and slan+ non-classical monocytes as two monocytic subpopulations with contrasting properties. Both types of monocytes even showed an inverse correlation in our study. Blood monocytes can be divided into classical (CD14highCD16−), intermediate (CD14highCD16+), and non-classical (CD14low/negCD16+) subpopulations. These subsets show transcriptomic differences that translate into specialization and different functions [46,47]. CD16+ monocytes can be further divided into slan-negative and slan+ subpopulations, the latter representing non-classical monocytes [48,49]. While being of monocytic origin, slan+ cells may either rapidly acquire DC functions or differentiate into macrophages [11]. Non-classical monocytes have been regarded as a pro-inflammatory population exhibiting tumor-killing properties [50]. In the context of malignant melanoma, CD16+ non-classical monocytes were shown to be crucial for ICI therapy, since they mediated the killing of regulatory T cells via a CTLA-4 (cytotoxic T lymphocyte-associated antigen 4)-specific mAb [51]. Slan+ monocytes can activate NK cells via IL-12, and the crosstalk between slan+ cells and NK cells improves differentiation of naïve CD4+ T lymphocytes into interferon (IFN)-gamma-producing Th1 cells [52]. In the current study, no obvious differences were found between patients with progression and short-term PFS, both for the baseline numbers of slan+ non-classical monocytes and the MDC/PDC sum. However, slan+ non-classical monocytes and the MDC/PDC sum better correlated with long-term survival (PFS ≥ 12months) than the NLR and could therefore be useful predictive markers. Interestingly, an inverse correlation could be observed between neutrophils and slan+ non-classical monocytes, and neutrophils and DC in this study: the higher the neutrophil counts, the lower were the amounts of both slan+ non-classical monocytes as well as the MDC/PDC sum, respectively. Some of the NSCLC patients had very low amounts of blood DC, which might contribute to their disturbed immune functions and poor prognosis. NSCLC patients have a significantly lower percentage of blood DCs than healthy donors [20,53]. The paucity of activated CD103+ DC in melanoma lesions has been discussed to limit ICI therapy efficacy [54]. Otherwise, a DC gene signature was strongly associated with improved patients’ OS in NSCLC patients undergoing Atezolizumab therapy (PD-L1 blockade) [55]. DC counts and their expression of coinhibitory molecules, such as PD-L1, can affect therapy response and patients’ survival; patients undergoing ICI monotherapy and exhibiting a higher PD-L1/CD274 expression of DC subtypes and monocytes, respectively, showed a significantly poorer survival [56]. Understanding and modulating DC counts and functional activity might help to improve the efficacy of T cell-centric immunotherapies in tumor patients [57].

In metastatic NSCLC, PD-L1 expression in tumor tissues is associated with a benefit from ICI therapy (KEYNOTE-024 trial) [58]. This observation could be confirmed in this study regarding the tumor lesions with high PD-L1 expression (≥50%). Furthermore, we found female sex to be an independent risk factor for a poor response to ICI therapy, an observation shared by Conforti et al. in a meta-analysis with patients of different tumor histotypes [59]. Male and female cancer patients have been discussed to respond in a different way to immunotherapies, regardless of the tumor histological type, the type of treatment, or the setting of therapy [59]. In animal studies, PD-1/PD-L1 expression might even be modulated by sex hormones [60]. In female study participants, a higher rate of hyperprogression was observed by Kanjanapan and coworkers [61]. Olingy et al. described a lower frequency of CD33high monocytes in the blood of female NSCLC patients and discussed a link to a reduced responsiveness to ICI therapy [25]. Comparing female and male patients in our cohort, female patients had a lower PD-L1 tumor expression, were more often never-smokers, and had more metastases. With respect to blood immune cells, female patients had lower baseline NK cell numbers, as already described earlier for an age-matched control group and NSCLC patients of different tumor stages [20]. Intriguingly, lower NK cell numbers correlated with worse PFS in this study.

Despite notable clinical responses, basic and clinical studies are still required to investigate the exact mechanism of immune checkpoint inhibitor immunotherapy and to improve the appropriate selection of patients. Identifying low percentages of both slan+ non-classical monocytes and DC as well as a high NLR and high percentages of HLA-DR^low^ monocytes as risk factors for patients’ response to a combined immune/chemotherapy, this study extends our knowledge on biomarkers and pathophysiological causes of a therapy resistance.

## 5. Conclusions

Adverse factors, which highlight NSCLC patients with primary resistance to a combination therapy of ICI and chemotherapy, are low baseline frequency of slan+ non-classical monocytes, a low sum of MDC/PDC, a high NLR, or high amounts of HLA-DR^low^ MDSC. Patients without any of the four risk factors had the best outcome, while patients with three or four risk factors had the shortest PFS in this study. A longer PFS could also be found for patients with ≥50% PD-L1 expression in tumor lesions, for male patients, and for those with baseline NK cell numbers ≥200 cells/µL blood. Understanding tumor-induced systemic immune cell abnormalities will lead to an improved risk evaluation of cancer patients and provide the rational for novel therapeutic options.

## Figures and Tables

**Table 1:** Patient characteristics and therapy response of patients. Patients were grouped by cancer histotype to show different therapy strategies.

Parameters	AC	SqC	NSCLC Other Than AC and SqC
Number	56	27	7
Age, median (IQR)	64 (15)	67 (8)	59 (17)
Sex			
Male, *n* (%)	35 (62.5)	25 (92.6)	4
Female, *n* (%)	21 (37.5)	2 (7.4)	3
ECOG			
0	34 (61.4)	14 (51.85)	3
1	22 (38.6)	13 (48.15)	4
2	0	0	0
PD-L1 expression, *n* (%)			
<1%	25 (44.6)	12 (44.4)	3 (42.9)
1–49%	18 (32.1)	9 (33.3)	2 (28.6)
≥50	11 (19.6)	6 (22.2)	2 (28.6)
missing	2		
Smoker status			
- Never-smoker	11 (19.6)	1 (3.7)	0
- Smoker	45 (80.4)	26 (96.3)	7 (100)
Metastases			
<3, *n* (%)	26 (46.4)	17 (63)	1 (14.3)
≥3, *n* (%)	30 (53.6)	10 (37)	6 (85.7)
Brain and/or liver metastases	16 (28.6)	6 (22.2)	3 (42.9)
Therapy setting:	Carboplatin	Carboplatin	Carboplatin
Chemotherapy	+ pemetrexed (TTF-1-pos.) or + nab-Paclitaxel (TTF-1neg.)	+ nab-Paclitaxel	+ nab-Paclitaxel
Therapy setting:	Pembrolizumab	Pembrolizumab	Pembrolizumab
ICI + others	or (if liver metastasis) Atezolizumab + Bevacizumab		
Radiatio before ICI, *n* (%)	6	3	1
Radiatio after ICI, *n* (%)	9	7	1
Patients with tumor recurrence, *n* (%)	10 (17.8)	7 (25.9)	1
Patients with primary advanced state, *n* (%)	46 (82.1)	20 (74.1)	6
Clinical response, *n* (%)			
- Progression/Discontinuation	14 (25)	8 (29.6)	1
- Disease stabilization	10 (17.9)	2 (7.4)	0
- Partial/complete remission	32 (57.1)	17 (63)	6 (85.7)

**Table 2:** Blood immune cell parameters at baseline and at third cycle of ICI/chemotherapy. The patients were grouped into progress/discontinuation and clinical response (stabilization of disease, or partial/complete remission). Median and interquartile range (IQR) are given.

Parameters	Baseline Values			Third Cycle Values		
	Progressive Disease/ Discontinuation	Clinical Response	*p*-Value	Progressive Disease/ Discontinuation	Clinical Response	*p*-Value
*n*	23	67		16	66	
Leukocyte counts (cells/μL)	11,000 (4600)	8910 (5550)		10,250 (7620)	7535 (4780)	0.004
Neutrophil counts (cells/μL)	8170 (5560)	6120 (4960)	0.016	7770 (7930)	4845 (3805)	0.002
Lymphocyte counts (cells/μL)	1890 (1250)	1630 (690)		1445 (1074)	1460 (1160)	
T cells (cells/μL)	1142 (695)	1086 (646)		1043 (826)	1109 (878)	
B cells (cells/μL)	209 (239)	109 (103)		160 (212)	75 (58)	
NK cells (cells/μL)	188 (396)	268 (276)		202 (340)	235 (196)	
NLR	4.54 (5.26)	3.88 (4.19)		6.77 (5.74)	3.46 (3.12)	0.006
Monocytes (cells/μL)	840 (340)	660 (280)		807 (352)	710 (423)	
CD16+ monocytes (% of monocytes)	9.3 (8.6)	13 (7.3)		10.2 (6.8)	14.6 (8.9)	
Slan+ monocytes (% leukocytes)	0.16 (0.32)	0.26 (0.54)		0.13 (0.21)	0.32 (0.52)	0.014
HLA-DR^low^ MDSC (% of monocytes)	7.9 (22.1)	6.9 (13.4)		11.4 (17.1)	6.65 (8.7)	0.026
CD1c^+^ MDC (% of leukocytes)	0.062 (0.074)	0.105 (0.091)	0.04	0.070 (0.063)	0.162 (0.143)	<0.001
CD141^+^ MDC (% of leukocytes)	0.004 (0.005)	0.007 (0.006)	0.022	0.004 (0.004)	0.008 (0.006)	0.001
PDC (% of leukocytes)	0.067 (0.068)	0.093 (0.092)		0.051 (0.047)	0.116 (0.134)	0.011
Sum of MDC/PDC (% of leukocytes)	0.142 (0.167)	0.198 (0.162)	0.043	0.149 (0.142)	0.313 (0.268)	0.001

**Table 3:** Relationship between baseline blood immune cell parameters with patients’ survival (3A PFS; 3B OS). Data of univariate prognostic factor analysis is provided, with estimated mean of survival ± standard error, hazard ratios (HR) with 95% confidence interval (CI) and *p* values.

3A	Cutoff	*n*	Kaplan–Meier PFS			Cox Regression, PFS		
			% Cen-sored	PFS (months)	*p*-Value	HR	95% CI	*p*-Value
Neutrophil counts(cells/μL)	≤10,000	67	50.7	16.7 ± 1.6	0.013	1	1.11–3.48	0.019
	>10,000	23	17.4	8.7 ± 1.6		1.98		
NLR	<6.1	61	52.5	16.9 ± 1.6	0.005	1	1.21–3.64	0.009
	≥6.1	29	20.7	9.3 ± 1.7		2.10		
NK cells (cells/μL)	<200	34	29.4	11.4 ± 2.05	0.030	1	0.32–0.97	0.038
	≥200	56	50.0	16.1 ± 1.67		0.56		
HLA-DR^low^ MDSC (% of monocytes)	<22	74	47.3	16.0 ± 1.52	0.027	1	1.05–3.69	0.036
	≥22	16	18.8	8.4 ± 1.98		1.96		
CD16+ monocytes (% of monocytes)	<10	28	28.6	10.2 ± 2.1	0.024	1	0.30–0.95	0.031
	≥10	60	48.3	16.4 ± 1.67		0.54		
Slan+ monocytes (% of leukocytes)	<0.25	35	17.1	6.97 ± 0.87	<0.001	1	0.18–0.58	<0.001
	≥0.25	52	59.6	19.3 ± 1.78		0.32		
Sum of MDC/PDC (% of leukocytes)	<0.14	31	19.4	7.1 ± 0.88	<0.001	1		
	≥0.14	59	54.2	17.9 ± 1.70		0.38	0.22-0.68	<0.001
3B	Cutoff	*n*	Kaplan-Meier OS			Cox Regression, OS		
			% censored	OS (months)	*p*-Value	HR	95% CI	*p*-Value
Neutrophil counts(cells/μL)	≤10,000	67	50.7	17.9 ± 1.47	0.012	1	1.14–3.53	0.016
	>10,000	23	17.4	10.5 ± 1.49		2.00		
NLR	<6.1	61	52.5	17.9 ± 1.49	0.008	1	1.18–3.53	0.011
	≥6.1	29	20.7	11.3 ± 1.55		2.03		
NK cells (cells/μL)	<200	34	29.4	13.1 ± 1.87	0.044	1	0.34–1.01	0.053
	≥200	56	50.0	17.4 ± 1.5		0.58		
HLA-DR^low^ MDSC (% of monocytes)	<22	74	47.3	17.2 ± 1.39	0.033	1	1.03–3.61	0.041
	≥22	16	18.8	10.3 ± 1.96		1.93		
CD16+ monocytes (% of monocytes)	<10	28	28.6	11.3 ± 1.72	0.030	1	0.31–0.97	0.038
	≥10	60	48.3	17.6 ± 1.52		0.55		
Slan+ monocytes(% of leukocytes)	<0.25	35	17.1	11.8 ± 1.38	<0.001	1	0.19–0.66	<0.001
	≥0.25	52	59.6	20.6 ± 1.8		0.35		
Sum of MDC/PDC (% of leukocytes)	<0.14	31	19.4	8.95 ± 0.81	<0.001	1	0.20–0.64	<0.001
	≥0.14	59	54.2	18.9 ± 1.55		0.36		

**Table 4:** Association of baseline blood immune cell parameters analyzed by Spearman’s rank correlation.

Baseline Blood Immune Cells	Correlation Coefficient	*p*-Value
Neutrophil number with monocyte count	0.420	<0.001
Neutrophil number with the frequency of HLA-DR^low^ MDSC	0.598	<0.001
Neutrophil number with the frequency of CD16+ monocytes	−0.477	<0.001
Neutrophil number with the frequency of slan+CD16+ monocytes	−0.599	<0.001
Neutrophil number with the frequency of MDC/PDC	−0.662	<0.001
HLA-DR^low^ MDSC with the frequency of MDC/PDC	−0.600	<0.001
HLA-DR^low^ MDSC with the frequency of CD16+ monocytes	−0.548	<0.001
HLA-DR^low^ MDSC with the frequency of slan+CD16+ monocytes	−0.440	<0.001

## Data Availability

The data well be available after publication on request.

## Supplementary Materials

The following supporting information can be downloaded at: https://www.mdpi.com/article/10.3390/cancers14153690/s1, Figure S1: Gating strategy for DC subpopulations; Figure S2: Gating stra-tegy for slan+ non-classical monocytes; Table S1: Receiver operating characteristic curve analysis for the prediction of long-term survival (PFS ≥ 12 months) by several single immune cell parameters; Table S2: Comparison of blood immune cells in female and male NSCLC patients.
