# Supplementary material for: Blood Immune Cell Biomarkers in Lung Cancer Patients Undergoing Treatment with a Combination of Chemotherapy and Immune Checkpoint Blockade
Source: Cancers (Basel). 2022 Jul 28;14(15):3690. doi: 10.3390/cancers14153690 (PMC9367406; doi:10.3390/cancers14153690)
Supplement: Supplementary file 1 [file cancers-14-03690-s001.zip › cancers-1809393-supplementary.pdf]

cancers

MDPI

Supplementary Materials

Blood Immune Cell Biomarkers in Lung Cancer Patients Undergoing Treatment with a Combination of Chemotherapy and Immune Checkpoint Blockade

Miriam Möller, Steffi Turzer, Georgi Ganchev, Wienke Andreas, Wolfgang Schütte, Barbara Seliger and Dagmar Riemann

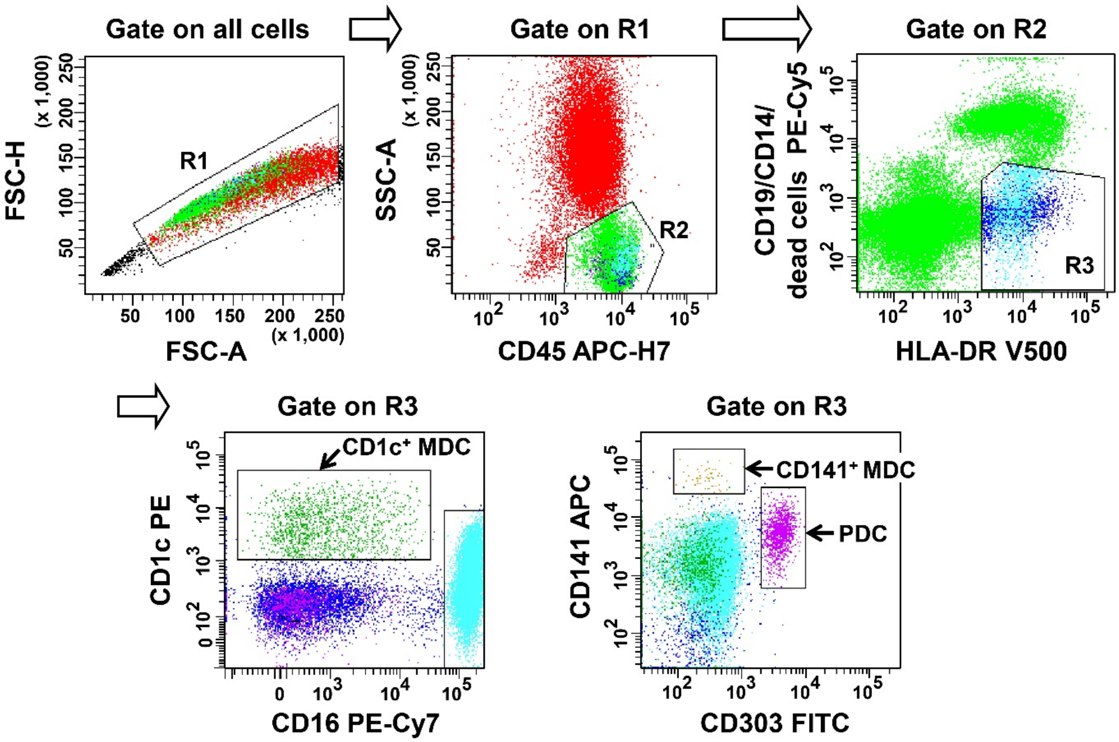

Figure S1. Gating strategy for DC subpopulations. Approximately 1x10E6 mononuclear cells were measured (storage of all cells). After doublet discrimination (R1), a gate on CD45++ mononuclear cells (R2) was used to exclude dead cells, CD14+ monocytes and B cells and to gate HLA-DR+ cells (R3). DC were subdivided into CD1c+ MDC, CD141+ MDC and CD303+ PDC. All 3 DC subpopulations were summarized as "sum of MDC/PDC".

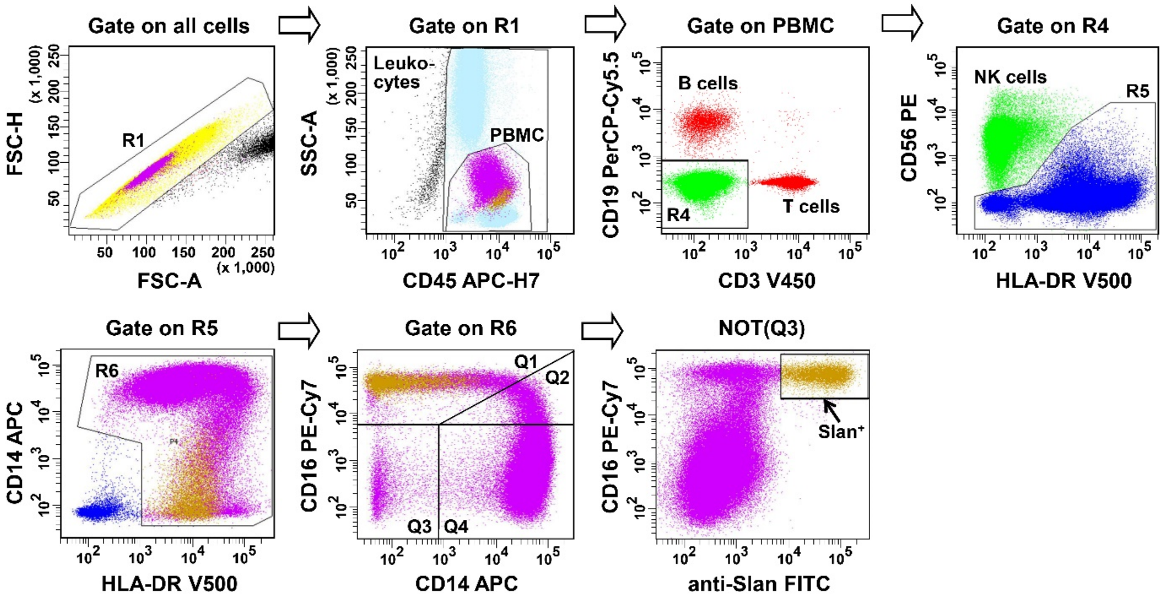

Figure S2. Gating strategy for slan+ non-classical monocytes. After doublet discrimination (R1), a gate on leukocytes and on CD45++ mononuclear cells (PBMC) as part of leukocytes is drawn. PBMC were used to exclude B cells and T cells (R4) and CD56+ NK cells (R5). Thereafter, HLA-DR-negative CD14-negative cells were excluded (R6). Total monocytes were defined as the sum of Q1+Q2+Q4. The last picture shows slan+ non-classical monocytes as part of CD16+ monocytes.

Table S1. Receiver operating characteristic curve analysis for the prediction of long term PFS (≥12months) by several single immune cell parameters (baseline values as 1, ICI-third cycle values as 2).

**Prediction Variable at baseline**	**Cutoff point**	**AUC**	**95% CI**	***p* value**
NLR_1	6.1	0.61		
HLA-DR^{low} monocytes_1 (% of monocytes)	22	0.59		
Slan+CD16+ monocytes_1 (% of leukocytes)	0.25	0.72	0.61-0.85	0.001
Sum of MDC/PDC_1 (% of leukocytes)	0.14	0.73	0.62-0.85	0.001
Prediction Variable at cycle 3 of ICI/chemotherapy	Cutoff point	AUC	95% CI	*p value*
NLR_2	6.1	0.75	0.64-0.86	<0.001
HLA-DR^{low} monocytes_2 (% of monocytes)	22	0.68	0.55-0.80	0.013
Slan+CD16+ monocytes_2 (% of leukocytes)	0.25	0.80	0.67-0.90	<0.001
Sum of MDC/PDC_2 (% of leukocytes)	0.14	0.82	0.71-0.91	<0.001

AUC indicates area under the ROC curve; CI, confidence intervall.

Table S2. Comparison of blood immune cells in female and male NSCLC patients. Median and interquartile range (IQR) are given.

	**Baseline values**		
	Female Patients	Male Patients	*p* value
n	26	64	
Age (IQR)	61 (18)	66 (11)	
Percentage of never-smokers	6 (23.1%)	6 (9.4%)	n.d.
≥3 metastases	17 (65%)	29 (45.3%)	n.d.
Neutrophil counts (cells/µl)	6155 (5983)	6655 (4980)	
Lymphocyte counts (cells/µl)	1570 (795)	1700 (985)	
T cells (cells/µl)	1074 (490)	1162 (799)	
B cells (cells/µl)	172 (170)	102 (112)	0.018
NK cells (cells/µl)	196 (172)	305 (313)	0.003
NLR	4.14 (5.12)	3.89 (4.26)	
Monocyte counts (cells/µl)	685 (243)	705 (380)	
HLA-DR^{low} MDSC (% of monocytes)	7.8 (18.4)	6.9 (12.9)	
CD16+ monocytes (% of monocytes)	13.2 (6.6)	12.3 (8.9)	
Slan+CD16+ monocytes (% leukocytes)	0.145 (0.43)	0.28 (0.473)	
CD1c+ MDC (% of leukocytes)	0.080 (0.098)	0.101 (0.095)	
CD141+ MDC (% of leukocytes)	0.004 (0.006)	0.007 (0.007)	0.025
CD303+ pDC (% of leukocytes)	0.068 (0.084)	0.092 (0.094)	
Sum of MDC/PDC (% of leukocytes)	0.188 (0.169)	0.197 (0.182)	
